# Free and Charitable Clinic Telehealth Adoption and Utilization During the COVID-19 Era: The North Carolina Experience

**DOI:** 10.1089/tmr.2023.0029

**Published:** 2023-08-03

**Authors:** Julie Ann Sakowski, Ashley Parks, Danielle Nunnery, Andrew Wear

**Affiliations:** ^1^ Department of Nutrition and Healthcare Management, Beaver College of Health Sciences, Appalachian State University, Boone, North Carolina, USA.; ^2^ Cratis D. Williams School of Graduate Studies, Appalachian State University, Boone, North Carolina, USA.

**Keywords:** free clinics, implementation, medically uninsured, telehealth, telemedicine, underserved populations

## Abstract

**Purpose:**

The emergence of the COVID-19 pandemic led health care systems and providers worldwide to rapidly adopt telehealth solutions to minimize risk and comply with isolation mandates. This article explores telehealth utilization trends in North Carolina (NC) free and charitable clinics—an ambulatory health care delivery setting where traditional third-party reimbursement policies are not a primary consideration.

**Methods:**

We surveyed NC free and charitable clinic administrators regarding clinic decisions to adopt an externally sponsored telehealth system, what services are provided by telehealth, clinic implementation processes, which populations used telehealth, how telehealth was incorporated into current clinic workflows, and perceptions of telehealth outcomes.

**Findings:**

Telehealth was rapidly adopted among free and charitable clinics after the COVID-19 outbreak. Reasons for implementing telehealth included the ability to continue providing services during a public health emergency and to increase access to patients. However, clinics report that telehealth utilization has dropped significantly since the initial pandemic surge. Patient and provider preferences for in-person services are a common reason cited for this drop. Free and charitable clinics report a strong interest in continuing to deliver services through telehealth. The majority reported continuing to offer telehealth services, but primarily as a supplement to in-person visits rather than as a replacement. They perceive that implementing telehealth has increased access to care but are less certain about the impact on cost of care and patient satisfaction. However, clinic administrators believe improvements in interoperability with other data systems, workflows, scheduling, and care delivery approaches are needed to achieve telehealth's fullest utilization.

**Conclusion:**

Telehealth can play a significant role in expanding access to services in the free and charitable clinic setting. However, continued refinements in the technology to facilitate integration with other systems and workflow processes are needed to reach its full potential.

## Introduction

Research has shown that telehealth has the capacity to increase access to physical and mental health services and reduce barriers to care, especially for underserved populations.^1,2^ Despite this evidence, telehealth adoption in the United States remained limited before 2020. Critical barriers to telehealth adoption have been shown to include cost, a lack of technical capacity, and uncertain or insufficient reimbursement.^3,4^

The public health emergency arising from the COVID-19 pandemic in the spring of 2020 produced a unique health care delivery environment. Infectious disease containment concerns coupled with expanded telehealth reimbursement policies prompted a dramatic increase in ambulatory telehealth visits by primary and specialty providers, producing a major shift in the trajectory of telehealth utilization.^5–8^ However, the continuation of this trend is uncertain as the surge of COVID-19 infections wanes and the public health emergency declaration is lifted.

An American Medical Association survey conducted in 2021 found that a major factor influencing physicians' decisions to continue offering telehealth services in the postpandemic future is uncertain reimbursement policies.^9,10^ *Although there is extensive evidence to describe telehealth uptake since COVID-19, little evidence exists on how telehealth has been implemented and integrated into work flows in ambulatory settings serving vulnerable populations, what services are being provided via telehealth and for which patient populations.*

### Background and significance of these data

The purpose of this study was to explore how telehealth use has changed since the height of the COVID-19 pandemic, what it is used for, and by whom in a unique category of providers: North Carolina (NC) free and charitable clinics.

The North Carolina Association of Free and Charitable Clinics (NCAFCC) is a nonprofit organization that supports and provides sustainable funding sources for 70 member clinics and 85 clinic sites across NC.

Member clinics are quite varied, differing in the scope of services provided, funding sources, religious affiliation, patient eligibility criteria, whether they accept Medicaid or other payments, and staffing models (e.g., proportion of paid versus volunteer providers and staff). The common denominator is that they provide care for the uninsured and underinsured in NC.^11^ In March 2020, NCAFCC offered a comprehensive telehealth platform free of charge to its members.

Since NC free and charitable clinics frequently do not bill for their services and had access to a telehealth technology platform free of charge, they do not face two common barriers for telehealth uptake: lack of resources for technology investment and insufficient or uncertain telehealth reimbursement rates. This study, therefore, provides insight into telehealth utilization patterns for serving uninsured and underinsured populations and the motivation behind those decisions when specific key financial constraints are absent or reduced.

## Materials and Methods

The Rogers Model of Diffusion outlines five steps integral to fully integrate any new technology into practice: dissemination, adoption, implementation, evaluation, and institutional uptake.^12^ This study was designed to collect exploratory data on NC free and charitable clinics' experiences and progress along this continuum. Since information on telehealth is widely available, and a single platform was offered to members, eliminating the need for product selection, this study did not focus on the dissemination stage.

Rather, the survey focused on the adoption, implementation, evaluation, and uptake stages by asking questions regarding clinic decisions to adopt the association-sponsored telehealth system or not; what services are provided by telehealth, clinic implementation processes, which populations used telehealth, how telehealth was incorporated into current clinic workflows, and perceptions of the system. The survey was pilot tested by a panel of health care management test readers for usability and face validity before dissemination (Supplementary Data).

Current NCAFCC member clinics were invited to participate in the study. Survey recruitment took place during virtual presentations at monthly regional clinic director meetings in September and October 2022 where the research team introduced the study and invited attendees to participate, offering a $25 gift card to complete the survey. After the meeting, an e-mail describing the study and a link to the online Qualtrics survey were sent to clinic directors. Directors were asked to forward the survey or complete it in collaboration with individuals who may have been more actively involved in the telehealth adoption decision-making and implementation process than the directors themselves.

Descriptive statistics were used to summarize survey response patterns. Open-ended responses were evaluated using thematic analysis and open coding using MaxQDA Analytics Pro 2022. A grounded theory approach was utilized to generate a summary of emerging themes through familiarization with the data, open coding, and identification of significant themes^13^ crucial to the participant's clinics experience implementing telehealth services. The researchers used the constant comparative method to develop axial codes—categories of themes emerging from the survey comments—and selective codes that identify the linkages among the axial codes to further classify themes.^14,15^

This study was reviewed and certified exempt by the Appalachian State University Institutional Review Board.

## Results

**FIG. 1.**
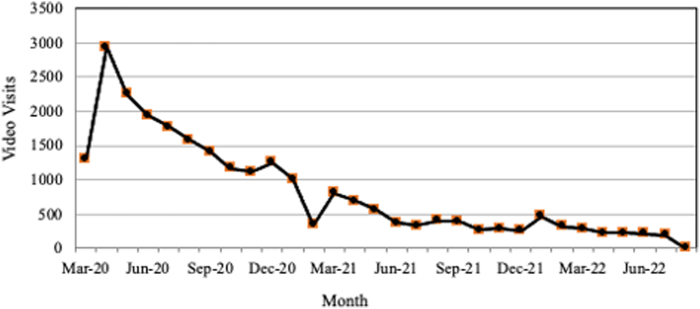
Telehealth video visits utilizing the NCAFCC-sponsored platform by March 2020 to August 2022. NCAFCC, North Carolina Association of Free and Charitable Clinics.

Of the 66 NCAFCC member clinics operating during the first half of 2020, 50 implemented the association-sponsored telehealth platform.^16^ Some of NCAFCC member clinics only offered dental, prescription medication provision, or referral services; thus, they may have chosen to decline the telehealth system because it was not appropriate for the services they offer. Figure 1 shows the sharp spike in telehealth usage in the spring of 2020, followed by a gradual decrease over time, consistent with telehealth usage trends reported across other health service sectors. Although all clinics reported a decline in telehealth video visit usage by late 2020, some clinics maintained a moderate level of telehealth usage for specific services through 2021 and 2022.

### Clinic characteristics

Usable survey responses were collected from 32 of the 57 NCAFCC member clinics providing medical and behavioral health services in 2022 (response rate 56%). As given in Table 1, survey nonrespondents tended to be smaller, offer part-time services, or be located in rural areas than those participating in the survey. It is important to note that rurality classifications are based on the main clinic address and may not reflect the rurality of the patient base served.

**Table 1.** Clinics Offering Medical or Behavioral Health Services in 2022

	Respondents (***n*** = 32), %	Nonrespondents (***n*** = 25), %
Location type		
Metropolitan	71	82
Micropolitan	29	9
Rural	0	9
Located in a medically underserved area	56	41
Located in a mental/behavioral health underserved area	82	91
Service hours		
30 or more hours per week	68	50
<30 h a week	32	50
Average number of annual patients medical visits (2021)	3512	1625
Average number of annual patient behavioral/mental health visits (2021)	638	311

### Adoption

Of the 32 respondents, 16 reported implementing the NCAFCC system. An additional six reported declining the association-provided system because they either already had a telehealth system in place or chose to implement another platform. Thus, 22 out of 32 (69%) respondent clinics implemented telehealth by mid-2020. An additional four respondents reported implementing another telehealth system after the initial association-sponsored telehealth rollout, but one ceased using telehealth postpandemic. As of the last quarter of 2022, 25 (78%) respondents reported using telehealth services in some form. The clinic characteristic data presented in Table 2 suggest that clinics that were more reliant on volunteer providers were less likely to adopt the new technology.

**Table 2.** Characteristics of Clinics Adopting Telehealth by 2020

Clinic characteristics	Adopted, %	Did not adopt, %
Urban	67	80
Micropolitan	33	20
Rural	0	0
Patient mean visit volume		
Medical services	3563	3429
Mental/behavior health	748	250
Mean # FT physicians	1.5	1
Mean # PT physicians	1.4	1.5
Mean # FT midlevel providers	1.9	1.8
Mean # PT midlevel providers	1.6	1.3
Mean physician volume hours	279	455
Mean midlevel volume hours	113	154

FT, full time; PT, part time.

**FIG. 2.**
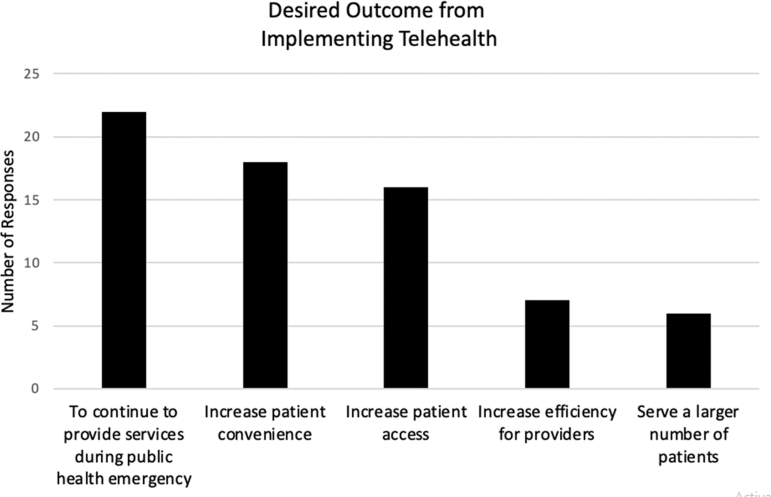
Reasons for implementing telehealth.

Objectives cited for adopting telehealth are shown in Figure 2. Respondents were allowed to report multiple reasons. The most common reasons were to continue providing services during the pandemic and to increase patient access and convenience. Of the six clinics that reported the *only* reason for implementing telehealth was to be able to continue to offer services during the pandemic, three continued to utilize telehealth beyond the height of the pandemic and reported they were still offering telehealth services in the last quarter of 2022.

Reasons for not adopting the association-sponsored telehealth platform include providers were not comfortable with the technology (*n* = 5), already had telehealth in place or chose another platform (*n* = 4), too difficult to use (*n* = 2), incompatibility with electronic health record (EHR; *n* = 2), lack of interest on part of clinic personnel (*n* = 1), lack of interest in some patients (*n* = 2), patient technology limitations (*n* = 2), and privacy concerns (*n* = 1).

### Telehealth implementation and adoption processes

The Advanced Consolidated Framework for Implementation Research (CFIR) provides a framework for evaluating elements associated with the success, failure, and outcomes achieved by implementing any intervention.^17^ The CFIR, widely used in implementation studies, consists of a list of contextual constructs grouped into five domains.^18,19^ We used the CFIR as our framework to describe how telehealth has been implemented and used in NC free and charitable clinics. Table 3 summarizes the key implementation processes undertaken by the clinics, grouped according to the CFIR contextual constructs.

**Table 3.** Implementation and Evaluation Activities

	Results		Respondent comments, %
Implementation team and resources			
Who led the implementation team	NCAFCC system users		
		Administrative management or personnel (e.g., clinic director)	35
		Clinical management or personnel (e.g., nurse manager, provider)	10
		IT personnel	5
		Interprofessional team	35
		Other	15
	Other system users	Administrative management or personnel (e.g., clinic director)	43
		Clinical management or personnel (e.g., nurse manager, provider)	21
		IT personnel	0
		Interprofessional team	36
		Other	0
Do users have an “expert” user to go to for assistance		Yes	60
		No	40
Technology needs			
Needed to add new technology to make telehealth work?		Yes	29
		No	71
Needed to “customize” system	NCAFCC system users	Yes	13
		No	87
	Other system users	Yes	29
		No	71
Overall perception of implementation process			
Experienced set up and implementation challenges	NCAFCC system users	Yes	9
		No	91
	Other system users	Yes	43
		No	57
Training			
Ease of learning	NCAFCC system users	Not easy	10
		Neutral	45
		Easy	45
	Other system users	Not easy	29
		Neutral	29
		Easy	42
How long was training?	NCAFCC system users	1 h or less	29
		Several hours	42
		1 day or more	29
	Other system users	1 h or less	29
		Several hours	43
		1 day or more	28
How was user training provided?	NCAFCC system users	In person/webinar	88
		Self-paced online modules	12
	Other system users	In person/webinar	100
		Self-paced online modules	0
Evaluation and refinement			
Did clinic perform an evaluation of the telehealth platform after implementation		Yes	52
		No	48
Made refinements after implementation		Yes	17
		No	83

IT, Information Technology; NCAFCC, North Carolina Association of Free and Charitable Clinics.

#### Implementation team

Clinics varied with who they chose to lead their telehealth implementation efforts. Efforts were led either by administrative management or personnel (35% for the NCAFCC-sponsored system) or an interdisciplinary team comprising individuals holding different roles across the clinic (35% for the NCAFCC-sponsored system). Almost two-third of the clinics report that someone, either within their organization or easily available outside the organization, has the experience and familiarity with the telehealth system to become an “expert” user that others can rely on when questions or issues arise.

#### Set up and implementation

Although many clinics reported they needed to purchase additional technology to perform telehealth services such as cameras, headsets, additional computers, and tablets for patient use, most report that they did not need to “customize” the telehealth system and were able to begin using the system “out of the box.” Configuration activities that were reported included modification of templates, adding the ability to create broadcast texts, disabling unnecessary billing features, and incorporation of documentation and subtitles for non-English speakers.

User training usually lasted several hours and was conducted in person or through a live webinar with in-house–created content. Forty-five percent of respondents who adopted telehealth rated the system as easy or very easy to learn, whereas only 10% rated it as difficult or somewhat difficult.

#### Integration with existing workflows

The primary challenge clinics reported during the initial implementation was integrating telehealth systems with their existing technology. For example, clinics reported challenges linking appointment-scheduling systems with telehealth and incorporating clinical notes into the EHR, requiring duplicative documentation. Clinics report overcoming these difficulties by altering their check-in and scheduling processes or switching telehealth platforms to better integrate them with their EHR system. Overall, 54% of respondents rated their transition to telehealth in their clinic as easy or somewhat easy, whereas 21% rated it as difficult or somewhat difficult.

### Telehealth use

Like the transition to remote work experienced across the economy since 2020, the rise in telehealth has provided greater flexibility in care delivery locations. Seventy-five percent of respondents reported that their providers were conducting telehealth visits, both from the clinic and at home. Twenty-one percent reported that providers were conducting telehealth visits only from the clinic, whereas only one clinic reported that their providers were solely conducting telehealth visits from home.

Clinics reported that patient use of telehealth was constrained not only by access to wifi, but also by the availability of devices needed to conduct telehealth visits, such as smartphones, cameras, and computers. Clinics reported that their patients connected to telehealth visits from home, work, “in the parking lot,” “anywhere they can get Wi-Fi,” and from within the clinic itself to connect with offsite providers. If they had technology, patient use of telehealth was also constrained by data plan expenses and limitations. For example, one clinic reported their patients “refused telehealth visits” because clients “with limited resources or minutes would not ‘waste’ 15–30 minutes on a telehealth call.”

This barrier was not unique to the free and charitable clinics in our study. Studies have suggested that audio-only telephonic telehealth visits instead of the more “higher-tech” audio–video may be a response to overcoming these constraints in uninsured, Medicaid, and other underserved populations.^20^ However, the quality of audio-only telehealth and the impact on health equity are still open to debate.^21^

Three-quarters of the clinics in our study reported currently offering telehealth encounters using both audio–visual interactive visits and audio-only calls (*n* = 17). In addition, three of these clinics utilized remote patient monitoring, and one used asynchronous communication such as text messaging and e-mail for telehealth delivery. Twenty-two percent (*n* = 6) are currently offering telehealth using only audio-only calls, and one only uses audio–visual interactive visits.

One respondent did not identify the current telehealth modality used. Table 4 provides additional context about the clinics that have continued to use telehealth beyond the pandemic. We found no association between continued telehealth utilization and who led the telehealth implementation team, clinic perceptions of ease of telehealth use, and the local operating environment.

**Table 4.** Association Between Continued Use of Telehealth Beyond COVID-19 and Organizational Characteristics

Measure	Currently use, %	Currently do not use, %	Fisher's exact test ***p***
Implementation team lead			0.247
Senior administrator(s)	10	5	
Administrative staff	4	5	
Clinical leadership	5	0	
IT staff	5	0	
Interdisciplinary team	62	5	
Perceived ease of telehealth use			1.0
Easy to use	20	4	
Neutral	12	0	
Not easy	54	12	
Setting			1.0
Metropolitan area	68	4	
Micropolitan area	28	0	
Primary care shortage area			1.0
Yes	72	4	
No	24	0	
Mental/behavioral health care shortage area			1.0
Yes	76	4	
No	20	0	

Free response questions in the survey enabled respondents to expand on patient and provider barriers to accessing and utilizing telehealth services. Table 5 presents the selected comments sorted by the identified theme and the wording of the survey question that generated the comment.

**Table 5.** Thematic Analysis of Survey Questions: Selected Comments by Theme and Survey Question

Theme—selective codes	Axial code—category	Open code—subcategory	Survey question	Comment
Barriers	Patient barriers (customer side barriers)	Patient preference	Q30. Think about the initial telehealth set up process. Did you experience any challenges getting the system to operate properly for your clinic?—Yes,—please briefly describe:	“Patient buy in, especially our technology challenged or older patients.”
		Patient technology access/issues	Q30. Think about the initial telehealth set up process. Did you experience any challenges getting the system to operate properly for your clinic?—Yes,—please briefly describe:	“Our patient population is very economically deprived, with 89% surviving below the FPL. They didn't have data plans on their phones; the pandemic shut down 100% of the guest wifi spots in our community. We had to purchase guest wifi for our location and stage a wifi access campaign in our community to enable patients to utilize telehealth.”
			Q3. Why did your organization choose to not implement the system made available by NCAFCC? (select all that apply)	“Our patients do not always have access to internet or smart devices or data for their devices.” “limited cell phone and wifi access for our patients”
	Process barriers	Workflow	Q3. Why did your organization choose to not implement the system made available by NCAFCC? (select all that apply)	“Patients get their prescriptions refilled as part of their visit so they must come into the clinic.”
			Q12. Why do you believe the amount of telehealth services your organization offers has declined since the COVID-19 pandemic?	“Hands on visits are ideal after a years' time”
				“Need for services (such as point of care testing) that can only be offered in person” “We used the system ‘out of the box’ with virtually no customization.”
				“Quality of clinical care—some of our clinical outcomes really dropped when patients did not come onsite”
				“We wish to obtain vitals.”
	Provider and staff barriers (provider side barriers)	Provider and staff knowledge	Q30. Think about the initial telehealth set up process. Did you experience any challenges getting the system to operate properly for your clinic?—Yes,—please briefly describe	*Staff were “green” with technology. It took a great deal of support and encouragement to get RNs and clinical staff to assume responsibility for telehealth visits with their patients*
		Provider and staff preference	Q3. Why did your organization choose to not implement the system made available by NCAFCC?	“Primary provider used another platform and preferred to use it”
			Q12. Why do you believe the amount of telehealth services your organization offers has declined since the COVID-19 pandemic?	“During the COVID-19 pandemic, our provider lived in Hawaii. We have replaced her with 4 on-site providers.”
			Q15. Why did your organization choose to not use the system after it was implemented?	“Provider unease with telehealth and lack of patient interest.”
		Organizational capacity	Q3. Why did your organization choose to not implement the system made available by NCAFCC?	“Not enough capacity to roll out at the time.”
Satisfiers and dissatisfiers	Satisfiers	Functionality and interoperability	Q43. What modifications or configuration changes did your organization make to the system?	“We used the system ‘out of the box’ with virtually no customization.”
	Dissatisfiers	Functionality and interoperability	Q30. Think about the initial telehealth set up process. Did you experience any challenges getting the system to operate properly for your clinic?—Yes,—please briefly describe:	“Athena telehealth was not as intuitive, and we had to create a few work arounds.”
…				“Technology platforms themselves often didn't have the capacity for the entire world to turn virtual overnight; there were often system glitches during the first six months especially.”
				“None of the telehealth technology effectively integrated with EMRs, so everything required double charting/data entry.”
				“Wish it was integrated with AthenaHealth; however, Athena has their own telehealth.”
			Q43. What modifications or configuration changes did your organization make to the system?	“We struggled with our Spanish speaking patients knowing what the prompts were telling them to sign in. they also struggled with getting a pin”

EMRs, electronic medical records; FPL, federal poverty line; RNs, registered nurses.

Several respondents commented on the process barriers related to data capture and essential clinical processes, such as vital signs and point-of-care testing, which require in-person visits. Without the ability to complete vital signs or on-site point-of-care testing, clinics struggled to meet their quality metrics and conduct active chronic disease management. Collectively, these open-ended responses speak of the challenges of implementing telehealth services during the COVID-19 pandemic and highlight the unique challenges and resilience of free and charitable clinics.

Consistent with industry-wide trends, respondents universally reported that their use of telehealth declined since the height of the pandemic, as shown in Figure 1.^8^ Table 6 shows that the volume and variety of services offered through telehealth by clinics have decreased slightly since the height of the pandemic, whereas behavioral and mental health services have remained constant across the majority of clinics with 21 out of 23 clinics (91%) originally offering primary care and care coordination during the pandemic continuing to do so in Fall 2022. Also, 100% (12 out of 12) of clinics offering mental health services virtually during the pandemic reported they were continuing to do so.

**Table 6.** Number of Clinics Offering Services Through Telehealth, by Type of Service

	Offering in 2020–2021	Currently offering as of Fall 2022
Medical management:	22	21
Chronic disease management	23	21
Specialty care	7	6
Mental/behavioral health	12	12
Acute care/urgent care/same day	13	11
Preventive care or primary care	23	21
Care coordination	16	14
Hospital or ED follow-up care	10	7
Acute care/inpatient	4	3
Other	1	1

ED, emergency department.

### Patient telehealth preferences

Clinics overwhelmingly reported a perception that their patients preferred in-person visits but would accept a telehealth visit if it could be scheduled sooner (88%). None of the respondents indicated that they perceived their patients preferred telehealth; only one reported that their patients refused telehealth, and two indicated that their patients were indifferent between telehealth and in-person visits. Clinics reported that although telehealth may reduce transportation barriers associated with in-person visits, their patient population may experience other barriers to utilizing telehealth.

In addition to the clinic perception that patients have a general preference for inpatient encounters, respondents identified access to technology and broadband, expenses incurred by using mobile data plans for telehealth visits, limited digital literacy, interpretation/language difficulties communicating through telehealth, and a general lack of awareness of telehealth availability as barriers to telehealth utilization and uptake by their patients.

### Telehealth use and outcomes

**FIG. 3.**
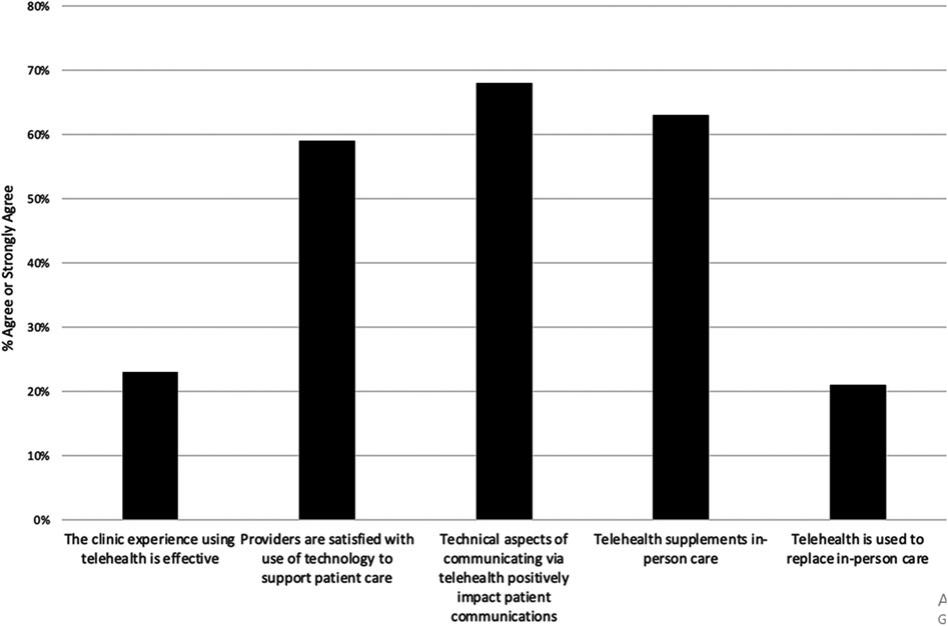
Clinic perceptions of telehealth implementation and use.

**FIG. 4.**
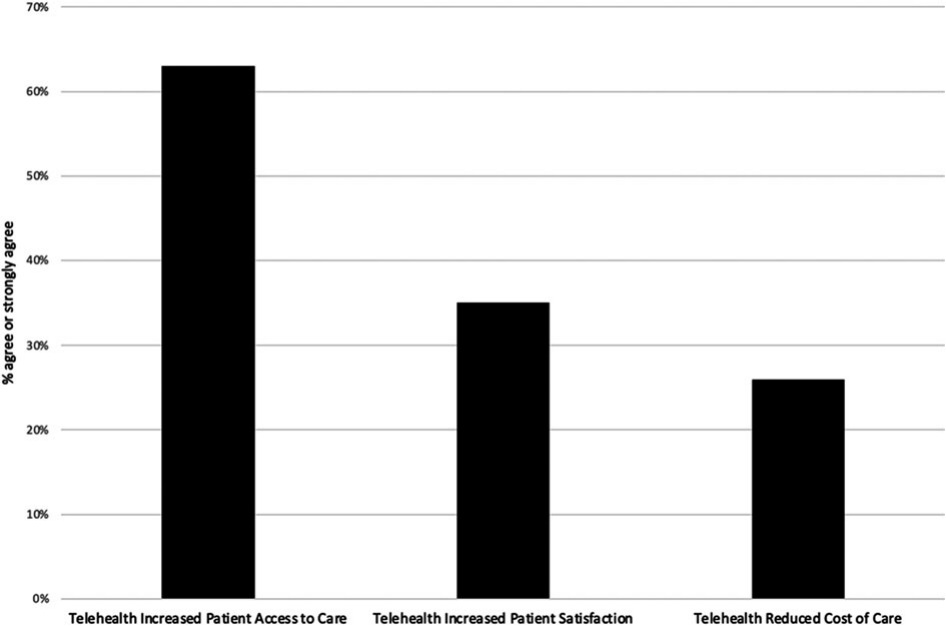
Clinic perceptions of telehealth outcomes.

Figure 3 shows respondents' perceptions of their clinic's experiences with the implementation and use of telehealth. Figure 4 shows clinic perceptions of the outcomes experienced using telehealth.

### Continued use: uptake

Ninety percent of respondents reported that they were interested in offering telehealth services in the future, even if they were not currently offering telehealth services. The reasons cited include increasing patient access (92%), reducing unnecessary patient costs (81%), increasing patient satisfaction (54%), virtual care has proven to be clinically and/or operationally effective (50%), and providing more comprehensive care (26%). However, the respondents identified technological and workflow refinements that may facilitate wider adoption and continued use.

Refinements to telehealth platforms and workflows suggested to increase usability and facilitate continued use include dedicating distinct appointment slots for telehealth versus in-person visits, addition of more staff to support digital workflows, developing best practices for check-in and check-out procedures, addition of a digital waiting room within the platform, additional telehealth training for all staff, and remodeling of clinic workspace to better support virtual care.

## Discussion

The objective of this study was to describe the telehealth adoption and utilization experience of free and charitable clinics, an under-represented sector of the health care system in telehealth utilization research. The clinics in this study were unique in that they received access to a free telehealth platform through the NCAFCC that reduced one of the most critical barriers to telehealth adoption: cost.

The findings in this study are consistent with the telehealth adoption and utilization trends observed across the United States in response to the emergence of the COVID-19 pandemic reported in the literature. Figure 1 shows that free and charitable clinics experienced a quick upsurge in telehealth utilization at the start of the pandemic in the United States, and that utilization increased, to a much smaller degree, during the various “infection surges” that occurred between the winter of 2020 and 2021.

However, the overall telehealth utilization rates dropped as the initial pandemic crisis eased. Thirteen of the clinics that initially implemented the association-provided “free” telehealth platform reported that they ultimately dropped that system and transitioned to another telehealth platform. This suggests that once in use, clinics saw value in offering services through telehealth, so much so that they were willing to pay for it. Clinics perceived that telehealth increased access to care, but clinic perceptions were mixed about the impact on the cost of care and patient satisfaction.

Our finding that respondents expressed an interest in continuing to offer telehealth services in the future, with modifications or under certain conditions, is consistent with the American Medical Association (AMA) survey of physicians practicing in general. Telehealth services are currently predominantly used as a supplement to in-person visits rather than as a replacement. Clinics report that their leadership is supportive of continued telehealth use, but one of the biggest challenges they currently experience is the linkage between the telehealth platform and existing systems, such as appointment scheduling and the EHR.

Workflow refinements that clinics believe would facilitate their continued use of telehealth included creating separate scheduling blocks for telehealth and in-person visits (52%), providing telehealth training for all clinic team members (48%), and creating a formal process for telehealth visit check-in/check-out (39%). Technological or resource refinements that would support future telehealth use included telehealth technology that provides a digital waiting room (26%), hiring additional staff to support telehealth workflows (26%), and remodeling office/examination room space to facilitate telehealth visits (22%).

### Limitations

This study provides exploratory insights into telehealth adoption and utilization for the uninsured and underinsured populations but has several limitations. Our findings may not be generalizable to telehealth implementation decision making in general, the use of telehealth in free and charitable clinics outside of NC, or clinics that are not members of the NCAFCC since the association provided a free telehealth platform. In addition, the low response rate from smaller, part-time, and rural clinics may cause our findings to under-represent their experiences. Therefore, our results may not be representative of the experience of all NCAFCC member-free and charitable clinics across the state.

This study explored the initial telehealth implementation processes during a crisis. Clinics reported that they implemented the telehealth system and transitioned to remote operation over a very short time. Thus, conclusions should not be inferred about implementation processes that might have occurred during “normal times.” This study does not allow us to draw conclusions on how telehealth implementation activities may have been formalized and what additional activities would have occurred had this not been done to rapidly transition operations during a crisis. This should be the focus of future research.

The target population for this survey was clinic personnel. Information on patient experiences with telehealth is based on the clinic personnel's perception of patient acceptance of telehealth and the barriers they experience. However, the findings from this study supplement existing literature describing the free and charitable clinic patient perspectives on the use of telehealth to provide a more complete picture.^22^ The exploratory technology adoption and process improvement focus of the survey provide limited information on patient characteristics utilizing telehealth. The lack of data from clinical sources prevents us from drawing in-depth conclusions about specific characteristics of patients using telehealth and the services best suited for telehealth. This should be a focus for future research.

## Conclusions

Telehealth plays a significant role in expanding access to services in the free and charitable clinic setting. However, continued refinements in the technology to facilitate integration with other systems and workflow processes are needed to reach its full potential. Although this study illustrated a strong desire on behalf of free and charitable clinics to initiate or continue the use of telehealth services going forward, these organizations, dedicated to serving vulnerable populations, face different barriers to telehealth adoption compared with hospitals and other ambulatory care settings, and represent a new frontier for the expanded use of telehealth services in the future.

The recent policy landscape has focused on barriers due to the lack of broadband service availability. We also need to examine the cost of accessing broadband, even when available, which may hinder the use of digital resources by low-income and vulnerable populations. Lastly, future research is needed to understand why patients and providers express a preference for in-person versus virtual visits. Identifying the perceived strengths and limitations of a telehealth visit is the first step toward improving and gaining acceptance. Virtual care delivery is a complete transformation of health care delivery. Best practices for telehealth encounters need to be developed, and both providers and patients need to be trained on how to deliver and receive care in this new model most effectively.

## Acknowledgments

We would like to acknowledge the support and cooperation we received from the North Carolina Association of Free and Charitable Clinics. This work could not have been completed without the assistance of Mark Scheerer, Director of Membership Engagement at NCAFCC. His facilitation efforts and insights were instrumental in completing this project and greatly appreciated.

## Authorship Contribution Statement

J.A.S., A.P., D.N., and A.W. contributed to the design and implementation of the research. J.A.S., A.P., and A.W. contributed to the analysis of the results and to the writing of the article.

## Institutional Review Board Approval

This study was reviewed and granted exempt from further review status (exempt) by the Appalachian State University Institutional Review Board.

## Supplementary Material

Supplemental data

## Abbreviations Used

CFIR: Consolidated Framework for Implementation Research
EHR: electronic health record
NC: North Carolina
NCAFCC: North Carolina Association of Free and Charitable Clinics
