# Supplementary material for: Free and Charitable Clinic Telehealth Adoption and Utilization During the COVID-19 Era: The North Carolina Experience
Source: Telemed Rep. 2023 Aug 3;4(1):215–26. doi: 10.1089/tmr.2023.0029 (PMC10457620; doi:10.1089/tmr.2023.0029)
Supplement: Supplemental data [file Supp_DataS1.docx]

Telehealth Implementation and Utilization by North Carolina Free and Charitable Member Clinics

Survey Instrument

Start of Block: Basic Questions

Q1 Does your clinic use an electronic health record (EHR)?

- no (4)
- yes; Allscripts (5)
- yes; Athena (6)
- yes; Cerner (7)
- yes; Epic (8)
- yes; GE Healthcare (9)
- yes; Meditech (10)
- yes; Nextgen (11)
- yes; Praxis (12)
- yes; other: (13) __________________________________________________

Q2 Did you implement the Updox system made available by NCAFCC?

- Yes (1)
- No (2)

Skip To: Q5 If Did you implement the Updox system made available by NCAFCC? = Yes

Display This Question:

If Did you implement the Updox system made available by NCAFCC? = No

Q3 Why did your organization choose to **not** implement the Updox system made available by NCAFCC? (select all that apply)

- Already had a telehealth platform in place (4)
- Not compatible with current EHR system in place (5)
- Providers not confident or comfortable with system and capabilities (6)
- Privacy or confidentiality concerns (7)
- Lack of interest on part of clinic personnel (8)
- Lack of interest on part of patients (9)
- Too difficult to use effectively (10)
- Other, please specify: (11) __________________________________________________

Q4 Has your clinic **ever** used telehealth to provide services to your patients?

- Yes (1)
- No (2)

Skip To: End of Block If Has your clinic ever used telehealth to provide services to your patients? = No

Q5 What was your desired outcome from implementing telehealth services? (Select all that apply)

- To continue to provide services during public health emergency shutdowns (1)
- Increase access for patients (2)
- Increase convenience for patients (3)
- Increase efficiency for providers (4)
- Serve a larger number of patients (5)
- Other, please specify: (6) __________________________________________________

Q6 Does your clinic **currently**use telehealth to provide services to your patients?

- Yes (1)
- No (2)

Q7 Approximately, what is the average number of patient visits your clinic currently serves per week, including both in-person and telehealth?**

________________________________________________________________

Q8 Approximately, how many telehealth visits does your clinic currently perform per week?

________________________________________________________________

Q9 Did your clinic provide telehealth services at any point during the COVID-19 pandemic?

- Yes (1)
- No (2)

Skip To: Q13 If Did your clinic provide telehealth services at any point during the COVID-19 pandemic? = No

Q10 Approximately, what percent of your clinic’s patient visits were performed using telehealth (audio or video) during the height of the COVID-19 pandemic (March 2020-March 2021)?

|  | 0 | 10 | 20 | 30 | 40 | 50 | 60 | 70 | 80 | 90 | 100 |
| --- | --- | --- | --- | --- | --- | --- | --- | --- | --- | --- | --- |

| Percent of Visits Conducted Via Telehealth () | 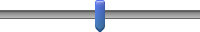 |
| --- | --- |

Q11 Has your clinic’s use of telehealth decreased since you first offered telehealth services during the COVID-19 pandemic?

- Yes (1)
- No (2)

Skip To: Q13 If Has your clinic’s use of telehealth decreased since you first offered telehealth services during... = No

Q12 Why do you believe the amount of telehealth services your organization offers has declined since the COVID-19 pandemic?

- Provider preference for in person visits (4)
- Provider scheduling efficiency (5)
- Patient preference for in person visits (6)
- Patient technology challenges (7)
- Other (8) __________________________________________________

Q13 How does your clinic’s *current* telehealth **cancellation or no-show rates** compare to in-person visits?

- Telehealth missed visit rates are significantly higher (4)
- Telehealth missed visit rates somewhat higher (5)
- No difference they are about the same (6)
- In-person missed visit rates are somewhat higher (7)
- In-person missed visit rates are significantly higher (8)

End of Block: Basic Questions

Start of Block: Expert Users

Q40 Do clinic users have an ‘expert’ user to go to if they have questions about using the telehealth system?

- Yes (1)
- No (2)
- N/A - Not applicable - Clinic does not use telehealth (3)

End of Block: Expert Users

Start of Block: Telehealth Optimization

Q41 Did your clinic assess or review how Updox or your current telehealth system was operating after it was in use?

- Yes (1)
- No (2)

Q42 Did your clinic have to modify or re-configure Updox or your current telehealth system in any way for your particular clinic after you started using it?

- Yes (1)
- No (2)

Display This Question:

If Did your clinic have to modify or re-configure Updox or your current telehealth system in any way... = Yes

Q43 What modifications or configuration changes did your organization make to the system?

________________________________________________________________

________________________________________________________________

________________________________________________________________

________________________________________________________________

________________________________________________________________

Q44 Have you experienced any difficulties sharing data between Updox or your current telehealth and other information systems within your clinic?

- Yes - please specify the issues: (1) __________________________________________________
- No (2)

Q45 Are there any specific features or capabilities you would add to Updox that would make using the system easier or more efficient?:

________________________________________________________________

Q46 Are there any specific features or capabilities you would add to your current telehealth system that would make using the system easier or more efficient?:

________________________________________________________________

Q47 How are you currently measuring the value of telehealth in your organization? (select all that apply)

- Patient satisfaction (1)
- Access to care (2)
- Clinical outcomes and clinical quality (3)
- Practice operational efficiency and effectiveness (4)
- Clinician experience (5)
- Reimbursement/payment (6)
- Cost saving (7)
- Health equity (8)
- We currently do not measure the value of telehealth (9)
- Other (10)

End of Block: Telehealth Optimization

Start of Block: Use of Telehealth

Display This Question:

If If Why did your organization choose to not implement the Updox system made available by NCAFCC? (select all that apply) Text Response Is Not Displayed

Q48 From where have your providers conducted your telehealth visits? (select all that apply)

- Clinic (4)
- Home (5)
- Hospital (6)
- Other, please specify: (7) __________________________________________________
- Don't know (8)

Display This Question:

If If Why did your organization choose to not implement the Updox system made available by NCAFCC? (select all that apply) Text Response Is Not Displayed

Q49 Where are your patients typically located during their telehealth visits? (select all settings that apply)

- Home (10)
- In the clinic to connect with a provider at another location (11)
- Another clinic/distant site (12)
- Hospital (13)
- Other, please specify: (14) __________________________________________________
- Don’t know (15)

Q50 What aspects of care does your clinic *currently* provide via telehealth? (select all that apply)

- Treatment or therapy (4)
- Screening/assessments/diagnosis (5)
- Follow up care (post surgical, chronic care, post hospitalization) (6)
- Continuous monitoring (7)
- Intake or triage (8)
- Other, please specify: (9) __________________________________________________
- None of the above (10)

Q51 What services do you or your practice currently provide via telehealth and which services were offered via telehealth during the height of the COVID-19 pandemic (March 2020-March 2021)? (select all that apply)

|  | 2020-2021 (1) | Currently (4) |
| --- | --- | --- |
| Medical management (eg medication management) (4) |  |  |
| Chronic disease management (5) |  |  |
| Specialty care (6) |  |  |
| Mental/behavioral health (7) |  |  |
| Acute care/urgent care/same day (8) |  |  |
| Preventive care or primary care (9) |  |  |
| Care coordination (10) |  |  |
| Hospital or ED follow up care (11) |  |  |
| Acute care/inpatient (12) |  |  |
| Other, please specify: (13) |  |  |
| None of the above (14) |  |  |

Q52 Which of the following telehealth modalities does your clinic *currently* use to provide clinical care to patients? (select all that apply)

- Audio visual interactive telehealth visits (4)
- Telephone/audio only calls with patients (5)
- Asynchronous telehealth (6)
- Remote patient monitoring of a patient (7)
- Other telehealth, please specify: (8) __________________________________________________
- None of the above (9)

Q53 Which communication platforms does your clinic *currently* use to deliver telehealth services? (select all that apply)

- Audio only telephone visits (4)
- Zoom (5)
- Doximity video (6)
- EHR telehealth module or tools (7)
- Doxy me (8)
- Telehealth vendor (9)
- FaceTime (10)
- Patient portal (11)
- Microsoft teams (12)
- Texting (13)
- Skype (14)
- Remote patient monitoring tools (15)
- Asynchronous messaging app (16)
- Other, please specify: (17) __________________________________________________

Q54 On a scale of 1-5, with 1 being strongly disagree and 5 being strongly agree, rank your agreement with the below sentence. (Please drag the bar to the appropriate number)

|  | Strongly disagree | Slightly disagree | Neutral, Neither Agree nor Disagree | Slightly agree | Strongly agree |
| --- | --- | --- | --- | --- | --- |

|  | 1 | 2 | 3 | 4 | 5 |
| --- | --- | --- | --- | --- | --- |

| Technical aspects of communicating via telehealth positively impact the effectiveness and quality of the patient interactions with providers. () | 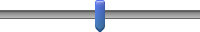 |
| --- | --- |

Q55 Which of the following, if any, do you perceive as barriers to your clinic’s patients using telehealth? (select all that apply)

- Limited patient access to technology (17)
- Limited digital literacy in patient mix (18)
- Limited patient access to broadband internet (19)
- Patient preferences for in-person visits (20)
- Limited patient access to data through their cellular plan (21)
- Limited patient awareness or understanding of insurance coverage for telehealth (22)
- Patient awareness of telehealth offerings (23)
- Lack of access to language/interpretation services (24)
- Lack of insurance (25)
- No barriers known (26)
- Limited access to community based resources (27)
- All of the above (28)
- Other, please describe: (29)

Q56 Which of the following, if any, do you anticipate being ongoing barriers or challenges to your organization offering telehealth? (select all that apply)

- Rollback of COVID 19 waivers, coverage, payment policies (4)
- Lack of insurance coverage of telehealth services (5)
- Low or no reimbursement (6)
- Technology challenges for my patient population (7)
- Licensure in additional states (8)
- Liability (9)
- Integration with EHR (10)
- Lack of technical support (11)
- Telehealth-specific workflows (12)
- Integration of additional technologies (13)
- Lack of guidelines for clinical appropriateness in telehealth (14)
- Low patient engagement (15)
- Clinician dissatisfaction/lack of buy-in (16)
- Cost of implementing or maintaining telehealth platform (17)
- Little or no buy-in from administrators or leadership (18)
- Lack of marketing for telehealth services (19)
- I do not anticipate barriers or challenges (20)
- Other, please describe: (21) __________________________________________________

Q57 On a scale of 1-5, with 1 being strongly disagree and 5 being strongly agree, rank your agreement with the following statements (Please drag the bar to the appropriate number for each).

|  | Strongly disagree | Slightly disagree | Neutral, Neither Agree nor Disagree | Slightly agree | Strongly agree |
| --- | --- | --- | --- | --- | --- |

|  | 1 | 2 | 3 | 4 | 5 |
| --- | --- | --- | --- | --- | --- |

| Patients have better access to care since our clinic started using telehealth () | 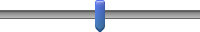 |
| --- | --- |
| Patients have higher satisfaction since our clinic started using telehealth () | 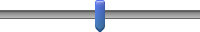 |
| Telehealth has decreased the cost of care () | 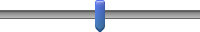 |
| Most of the telehealth visits replace in-person care () | 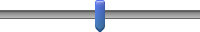 |
| Most of the telehealth visits supplement in-person care () | 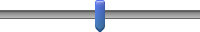 |
| The clinic’s leadership is motivated to continue to use telehealth () | 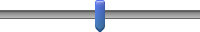 |

Q58 In general, how have your patients responded to being offered telehealth visits?

- They prefer telehealth visits (4)
- They are indifferent between telehealth and inperson visits (5)
- They prefer in person visits but will accept a telehealth visit if that can be scheduled sooner (6)
- They refuse telehealth visits (7)

Q59 Please share any comments you have regarding patient experience or response to telehealth visits:

________________________________________________________________

________________________________________________________________

________________________________________________________________

________________________________________________________________

________________________________________________________________

Q60 On a scale of 1-5, with 1 being very difficult and 5 being very easy, how would you rate your clinic’s transition to offering services via telehealth? (Please drag the bar to the appropriate number).

|  | Very difficult | Somewhat difficult | Neither easy nor difficult | Somewhat easy | Very easy |
| --- | --- | --- | --- | --- | --- |

|  | 1 | 2 | 3 | 4 | 5 |
| --- | --- | --- | --- | --- | --- |

| Ease of Transition () | 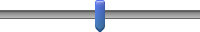 |
| --- | --- |

Q61 On a scale of 1-5, with 1 being very effective and 5 being very ineffective, how would you rate *your clinic’s experience delivering healthcare services using telehealth? (Please drag the bar to the appropriate number).*

|  | Very effective | Somewhat effective | Neither effective nor ineffective | Somewhat ineffective | Very ineffective |
| --- | --- | --- | --- | --- | --- |

|  | 1 | 2 | 3 | 4 | 5 |
| --- | --- | --- | --- | --- | --- |

| Ease of Use () | 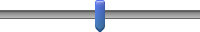 |
| --- | --- |

Q62 On a scale of 1-5, with 1 being strongly disagree and 5 being strongly agree, rank your agreement with the following statement. (Please drag the bar to the appropriate number for each).

|  | Strongly disagree | Slightly disagree | Neutral, Neither Agree nor Disagree | Slightly agree | Strongly agree |
| --- | --- | --- | --- | --- | --- |

|  | 1 | 2 | 3 | 4 | 5 |
| --- | --- | --- | --- | --- | --- |

| Overall, your clinic health care providers report they are satisfied with the use of technology to support patient care in your organization. () | 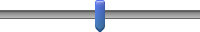 |
| --- | --- |

Q63 Which of the below would support improved telehealth-specific workflows? (select all that apply)

- Separate schedule blocks for in-person and telehealth visits (4)
- A formal process for patient check in and check out (5)
- Additional staffing resources to support workflows (6)
- Telehealth-specific training for other members of the care team (7)
- Technology that provides a digital waiting room (8)
- Better office/exam room space to facilitate telehealth visits (9)
- Other, please specify: (10) __________________________________________________

End of Block: Use of Telehealth

Start of Block: Future Use of Telehealth

Q64 What telehealth services does your practice or organization plan to offer in the future? (select all that apply)

- Medical management (4)
- Chronic disease management (5)
- Mental/behavioral health (6)
- Specialty care (7)
- Care coordination (8)
- Preventive care/primary care (9)
- Acute care or urgent care/same day (10)
- Hospital or ED follow up care (11)
- Acute care/inpatient (12)
- Other, please specify: (13) __________________________________________________
- We plan to decrease or eliminate telehealth offerings (14)
- None of the above (15)

Q65 Which of the following are reasons why you are interested in continuing care to provide telehealth? (select all that apply).

- Reduce patient barriers to access (13)
- Reduce unnecessary patient costs (time, travel, time off work, etc) (14)
- Increase patient satisfaction (15)
- Virtual care has been clinically effective (16)
- Virtual care has proven to be operationally effective (17)
- Increase professional satisfaction (18)
- Provide more comprehensive care (19)
- Other, please specify: (20) __________________________________________________
- Our clinic is not interested in continuing telehealth (21)

End of Block: Future Use of Telehealth

Start of Block: Use of the Updox Telehealth System

Q14 After implementing Updox, did your clinic use the system to provide patient services at any point in time?

- Yes (1)
- No (2)

Skip To: Q16 If After implementing Updox, did your clinic use the system to provide patient services at any point... = Yes

| Page Break |  |
| --- | --- |

Q15 Why did your organization choose to not use the Updox system after it was implemented?

________________________________________________________________

Q16 Has your clinic stopped using Updox and converted to another telehealth system?

- Yes- if yes, what system: (1) __________________________________________________
- No (2)

End of Block: Use of the Updox Telehealth System

Start of Block: Implementation Process

Title 6 The following questions are regarding your clinic's experience with the implementation and use of the Updox telehealth platform.

Q17 Who performed the installation/set up of Updox for your clinic (check all that apply)?

- Nurse manager (1)
- Clinic Director (2)
- IT support personnel (3)
- Other clinic administration personnel (4)
- A provider (5)
- Other clinical personnel (6)
- An Updox representative (7)
- Other (8)

Q18 Think about the initial Updox Set up process. Did you experience any challenges getting Updox to operate properly for your clinic?

- No (1)
- Yes, - please briefly describe: (2) __________________________________________________

Q19 Did you need to customize any Updox features for your organization?

- No (1)
- Yes - please specify which features: (2) __________________________________________________

Q20 To what extent were clinic providers involved with deciding how to customize the system?

- Not Involved (1)
- Somewhat Involved (2)
- Very Involved (3)

Q21 Did you need to add or purchase other technologies to be able to use Updox (i.e. new computers, servers, cameras, upgrade internet speeds)?

- No (1)
- Yes - What did you need to purchase or add? (2) __________________________________________________

End of Block: Implementation Process

Start of Block: Updox User Training

Q22 Who was trained on the Updox System? (check all that apply)

- All clinical care providers (1)
- Some clinical care providers - please explain: (2) __________________________________________________
- Clinic Managers/administrators (3)
- All clinic personnel (4)

Q23 On a scale of 1-5, with 5 being very easy and 1 being very difficult, how easy or difficult was the Updox system to learn how to use? (Please drag the bar to the appropriate number)

|  | Very difficult | Somewhat difficult | Neither easy nor difficult | Somewhat easy | Very easy |
| --- | --- | --- | --- | --- | --- |

|  | 1 | 2 | 3 | 4 | 5 |
| --- | --- | --- | --- | --- | --- |

| Ease of Use () | 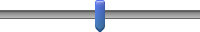 |
| --- | --- |

Q24 How were clinic personnel trained on using Updox? (select all that apply)

- In person (1)
- Via video conference (2)
- Online modules/ self paced tutorials (3)
- Other, please explain: (4) __________________________________________________

Q25 Did users receive Updox provided training materials, or training designed in-house by staff from your organization?

- Vendor supplied training (1)
- In house designed training (2)
- Both in house designed and vendor supplied training (3)

Q26 On average how long did your clinic users receive training for?

- > 1 day (more than a full work day) (1)
- 1 day (2)
- half a day (3)
- a few hours (4)
- an hour (5)
- less than an hour (6)

Q27 Did users receive hands-on practice simulating realistic scenarios during training?

- Yes (1)
- No (2)

Q28 Do clinic users have an ‘expert’ user to go to if they have questions about using the telehealth system?

- Yes (1)
- No (2)

End of Block: Updox User Training

Start of Block: Other System Implementation and Training

Q157 ***The following questions are regarding your clinic's experience with the implementation and use of a telehealth system other than Updox.***

Q29 When installing your telehealth system, who performed the installation/set up of telehealth for your clinic (check all that apply)?

- Nurse manager (1)
- Clinic Director (2)
- IT support personnel (3)
- Other clinic administration personnel (4)
- A provider (5)
- Other clinical personnel (6)
- A vendor representative (7)
- Other (8)

Q30 Think about the initial telehealth set up process. Did you experience any challenges getting the system to operate properly for your clinic?

- No (1)
- Yes, - please briefly describe: (2) __________________________________________________

Q31 Did you need to customize any telehealth features for your organization?

- No (1)
- Yes - please specify which features: (2) __________________________________________________

Q32 To what extent were clinic providers involved with deciding how to customize the system?

- Not Involved (1)
- Somewhat Involved (2)
- Very Involved (3)

Q33 Did you need to add or purchase other technologies to be able to use telehealth (i.e. new computers, servers, cameras, upgrade internet speeds)?

- No (1)
- Yes - What did you need to purchase or add? (2) __________________________________________________

Display This Question:

If After implementing Updox, did your clinic use the system to provide patient services at any point... != No

Q34 Who was trained on the telehealth System? (check all that apply)

- All clinical care providers (1)
- Some clinical care providers - please explain: (2) __________________________________________________
- Clinic Managers/administrators (3)
- All clinic personnel (4)

Display This Question:

If After implementing Updox, did your clinic use the system to provide patient services at any point... != No

Q35 On a scale of 1-5, with 5 being very easy and 1 being very difficult, how easy or difficult was the telehealth system to learn how to use?

|  | Very difficult | Somewhat difficult | Neither easy nor difficult | Somewhat easy | Very easy |
| --- | --- | --- | --- | --- | --- |

|  | 1 | 2 | 3 | 4 | 5 |
| --- | --- | --- | --- | --- | --- |

| Ease of Use () | 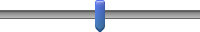 |
| --- | --- |

Display This Question:

If After implementing Updox, did your clinic use the system to provide patient services at any point... != No

Q36 How were clinic personnel trained on using the new telehealth system? (select all that apply)

- In person (1)
- Via video conference (2)
- Online modules/ self paced tutorials (3)
- Other, please explain: (4) __________________________________________________

Display This Question:

If After implementing Updox, did your clinic use the system to provide patient services at any point... != No

Q37 Did users receive provided training materials, or training designed in-house by staff from your organization?

- Vendor supplied training (1)
- In house designed training (2)
- Both in house designed and vendor supplied training (3)

Display This Question:

If After implementing Updox, did your clinic use the system to provide patient services at any point... != No

Q38 On average how long did your clinic users receive training for?

- > 1 day (more than a full work day) (1)
- 1 day (2)
- half a day (3)
- a few hours (4)
- an hour (5)
- less than an hour (6)

Display This Question:

If After implementing Updox, did your clinic use the system to provide patient services at any point... != No

Q39 Did users receive hands-on practice simulating realistic scenarios during training?

- Yes (1)
- No (2)

End of Block: Other System Implementation and Training
